# Potential benefits of adaptive intensity‐modulated proton therapy in nasopharyngeal carcinomas

**DOI:** 10.1002/acm2.13128

**Published:** 2020-12-18

**Authors:** Hideki Minatogawa, Koichi Yasuda, Yasuhiro Dekura, Seishin Takao, Taeko Matsuura, Takaaki Yoshimura, Ryusuke Suzuki, Isao Yokota, Noriyuki Fujima, Rikiya Onimaru, Shinichi Shimizu, Hidefumi Aoyama, Hiroki Shirato

**Affiliations:** ^1^ Department of Radiation Oncology Faculty of Medicine and Graduate School of Medicine Hokkaido University Sapporo Japan; ^2^ Department of Radiation Oncology Hokkaido University Hospital Sapporo Japan; ^3^ Global Station for Quantum Medical Science and Engineering Global Institution for Collaborative Research and Education (GI‐CoRE) Hokkaido University Sapporo Japan; ^4^ Department of Medical Physics Hokkaido University Hospital Sapporo Japan; ^5^ Department of Health Sciences and Technology Faculty of Health Sciences Hokkaido University Sapporo Japan; ^6^ Department of Biostatistics Faculty of Medicine Hokkaido University Sapporo Japan; ^7^ Department of Radiology Boston Medical Center Boston University School of Medicine Boston MA USA; ^8^ Department of Radiation Medical Science and Engineering Faculty of Medicine and Graduate School of Medicine Hokkaido University Sapporo Japan

**Keywords:** adaptive radiotherapy, dosimetric comparative study, intensity‐modulated radiotherapy, nasopharyngeal carcinoma, proton beam therapy

## Abstract

**Purpose:**

To investigate potential advantages of adaptive intensity‐modulated proton beam therapy (A‐IMPT) by comparing it to adaptive intensity‐modulated X‐ray therapy (A‐IMXT) for nasopharyngeal carcinomas (NPC).

**Methods:**

Ten patients with NPC treated with A‐IMXT (step and shoot approach) and concomitant chemotherapy between 2014 and 2016 were selected. In the actual treatment, 46 Gy in 23 fractions (46Gy/23Fx.) was prescribed using the initial plan and 24Gy/12Fx was prescribed using an adapted plan thereafter. New treatment planning of A‐IMPT was made for the same patients using equivalent dose fractionation schedule and dose constraints. The dose volume statistics based on deformable images and dose accumulation was used in the comparison of A‐IMXT with A‐IMPT.

**Results:**

The means of the D_mean_ of the right parotid gland (*P* < 0.001), right TM joint (*P* < 0.001), left TM joint (*P* < 0.001), oral cavity (*P* < 0.001), supraglottic larynx (*P* = 0.001), glottic larynx (*P* < 0.001), , middle PCM (*P* = 0.0371), interior PCM (*P* < 0.001), cricopharyngeal muscle (*P* = 0.03643), and thyroid gland (*P* = 0.00216), in A‐IMPT are lower than those of A‐IMXT, with statistical significance. The means of, D_0.03cc_, and D_mean_ of each sub portion of auditory apparatus and D_30%_ for Eustachian tube and D_0.5cc_ for mastoid volume in A‐IMPT are significantly lower than those of A‐IMXT. The mean doses to the oral cavity, supraglottic larynx, and glottic larynx were all reduced by more than 20 Gy (RBE = 1.1).

**Conclusions:**

An adaptive approach is suggested to enhance the potential benefit of IMPT compared to IMXT to reduce adverse effects for patients with NPC.

## 1 Introduction

The standard treatment for nasopharyngeal cancer (NPC) is radiotherapy and concomitant chemotherapy followed by adjuvant chemotherapy.^1^ Intensity‐modulated X‐ray radiotherapy (IMXT) has been used widely and is accepted as the treatment of choice for NPC.^2^, ^3^ However, adverse effects of IMXT with concomitant chemotherapy are still serious in a majority of patients and further improvements in radiation technology are warranted.^4^

In the IMXT of NPC, due to the changes in the body surface, weight loss, and the shrinkage of tumors during treatment, it is well known that tumor relapse and adverse‐effect rates may be increased by reduced doses to the clinical target volume (CTV) and excessive doses to the organs at risk (OARs).^5^, ^6^, ^7^, ^8^ Adaptive radiotherapy, in which treatment parameters are adjusted to conform to changes in anatomy during the irradiation, has been suggested to overcome these problems.^9^, ^10^ After the initial radiotherapy administration of 30–40 Gy using the initial plan, an additional 30–40 Gy is administered using an adapted plan which reflects the changes in anatomy. Encouraging improvements in loco‐regional control rates have been observed using adaptive radiotherapy, although advantages in overall survival were not obvious in a retrospective study.^10^

After the introduction of scanning proton beam technology, the usefulness of intensity‐modulated proton therapy (IMPT) has been shown to reduce the dose to various OARs for NPC and several dose comparative studies between IMXT and IMPT have been published.^11^, ^12^, ^13^, ^14^ Contrary to general expectations, Lewis et al. have pointed out that the superiority of IMPT to IMXT in reducing the dose to the OARs is not consistent among these studies.^14^ Widesott et al. even found opposite results for the mandible and larynx.^12^ IMPT with the adaptive approach (A‐IMPT) has recently been shown to be a promising future technology to enhance the dosimetric advantages compared to IMXT with the adaptive approach (A‐IMXT) for NPC.^15^ The spot size in the scanning beam for IMPT is becoming shaper than before^16^ and new technologies such as a short‐range applicator for treating superficial tumors^17^ are available with recent state‐of‐art apparatus. Treatment planning systems have been improved to make A‐IMPT practical in daily practice. As far as we were able to survey, however, no dosimetric studies have been reported comparing A‐IMXT with A‐IMPT for NPC.

Furthermore, recent treatment planning using precise imaging has made it possible to analyze detailed dose‐volume factors associated with ear disorders following radiotherapy in NPC^18^, ^19^ We have compared detailed dose volumetric statistics of OARs between A‐IMXT and A‐IMPT for hearing disorders. In the study, we investigated whether there are dosimetric advantages of A‐IMPT when compared to A‐IMXT for NPC by using simulation planning of IMPT for actual data of patients who have been treated with adaptive IMXT in our institution.

## 2 MATERIALS AND METHODS

### 2.1 Patient selection

Based on previous reports in the literature,^12^, ^14^ we assumed that the mean dose to the oral cavity in A‐IMPT would be 10 Gy (RBE = 1.1) lower than A‐IMXT with a standard deviation of 7 Gy (RBE = 1.1). To determine the differences in the mean dose to the oral cavity, the number of patients required to assure 80% power to detect differences in a two‐sided significance level of 1% was calculated by paired t‐test.^20^ The required sample size was calculated to be 10 by SAS® version9.4 (Cary, NC, USA).

There were 13 patients with NPC who were all treated with A‐IMXT (step and shoot method) and concomitant chemotherapy between April 2014 and July 2016 at Hokkaido University Hospital. Two patients were excluded because they received induction chemotherapy before IMXT with concomitant chemotherapy. One patient was excluded because of treatment by step and shoot IMXT in the initial part but by the volumetric arc therapy technique (VMAT) in the subsequent adaptive part. Finally, the remaining 10 patients were included in the dosimetric comparison. The characteristics of these 10 patients are shown in Table 1. The TNM classification and the clinical stage in Table 1 was according to the UICC TNM classification 7^th^ edition.

**Table 1:** Patient characteristics.

Patient number	Age	T stage*	N stage*	M stage*	Clinical stage
1	58	T1	N2	M0	III
2	66	T1	N2	M0	III
3	60	T1	N1	M0	II
4	67	T3	N1	M0	III
5	58	T1	N2	M0	III
6	69	T3	N1	M0	III
7	57	T3	N2	M0	III
8	59	T2	N1	M0	II
9	73	T2	N0	M0	II
10	63	T3	N1	M0	III

* UICC 7th edition.

### 2.2 Target volume, OARs, and dose prescription

This was a prospective clinical study (JCOG1015) based on an original study by Nishimura et al. in Japan with IMXT.^9^, ^21^ That protocol was based on practical standard IMXT in Japan for nasopharyngeal carcinoma. Our IMPT study was designed to be compatible with that protocol.

In the initial plan, the Gross tumor volume (GTV) consisted of both a primary lesion (GTV primary) and metastatic lymph nodes (GTV node). These were contoured using fused images of computed tomography (CT) and magnetic resonance imaging (MRI). The CTV primary and CTV node were implemented by adding a 1.0‐cm margin to the GTV and an additional margin for high‐risk patients accounting for microscopic extensions to the GTV primary and GTV node, respectively. The clinical target volumes in the initial plan (CTV46) consisted of the CTV46 primary, the CTV46 node and the prophylactic regional lymph node. The prophylactic lymph nodes were contoured according to international consensus guidelines.^22^ The planning target volume for the initial plan (PTV46) was implemented by adding an additional 3.0 mm as the setup margin to the CTV46.

For the adaptive plan, patients were rescanned by CT and MRI at the time when they were given 24 Gy in 12 fractions (24 Gy/12Fx) – 28 Gy/14Fx. The clinical target volume in the adaptive plan (CTV70) which consisted of the CTV70 primary and the CTV70 node was contoured using the rescanned CT. The planning target volume for the adaptive plan (PTV70) was produced by adding an additional 3.0‐mm as the setup margin to the CTV70.

The optic nerve, chiasm, pituitary gland, brain stem, spinal cord, parotid gland, temporomandibular (TM) joint, oral cavity, supraglottic larynx, glottic larynx, superior pharyngeal constrictor muscle (PCM), middle PCM, inferior PCM, cricopharyngeal muscle and thyroid gland were contoured according to the Charlotte et al. method.^23^ Middle and inner ear structures were contoured according to the Sun Y, et al. method.^18^ The volumes of middle ear (tympanic cavity and Eustachian tube), inner ear (cochlea and internal auditory canal), and mastoid air cells were contoured as components of the auditory apparatus. Skin was contoured by adapting a 5‐mm thickness as the depth of the skin from the surface according to the Kawamura study.^24^

A 3‐mm planning organs at risk volume (PRV) margin was added to the brain stem, optic nerve, optic chiasm, and spinal cord.

### 2.3 A‐IMXT planning

Pinnacle^3^ v9.0 (Phillips, Medical Systems, WI, USA) was used in the inverse treatment planning of IMXT using seven portals with the step and shoot technique.

In the actual treatment, 46 Gy in 23 fractions (46Gy/23Fx) was prescribed using the initial plan and 24Gy/12Fx was prescribed using the adaptive plan.

Dose constraints for OARs in the inverse planning with IMXT are shown in the left column of Supplementary Table S1 (Spinal cord PRV ≤50 Gy, Brainstem PRV ≤60 Gy, Optic nerves PRV ≤54 Gy, Optic chiasm PRV ≤54Gy). The plans were optimized to ensure 100% of the prescription dose to cover 95% of the volume of PTV46 and PTV70.

We created a plan to irradiate the PTV46 with 70Gy/35Fx on the initial CT (initial plan). The plan was created so that the dose regulation for the target and OARs would meet the criteria. Next, a plan to irradiate PTV70 with 70Gy/35Fx was created on the second CT (adaptive plan), and the dose regulation for the target/OARs was also created to meet the criteria. Finally, the dose in the initial plan was multiplied by 46/70 and the dose in the adaptive plan was multiplied by 24/70, and the sum was regarded as the dose to CTV and OARs.

### 2.4 A‐IMPT planning

VQA version 3.077 (Hitachi co ltd., Tokyo) was used in the inverse treatment planning of IMPT using three portals at 80°, 160°, and 200°. The dose distribution was calculated using the beam profile of the spot scanning dedicated proton beam therapy system, PROBEAT‐RT, (Hitachi, co ltd., Tokyo).

Relative biological effectiveness (RBE) was assumed to be 1.1 throughout the irradiated volume and we used 1 Gy (RBE = 1.1), as the unit of absorbed dose in the treatment planning. In the actual treatment, 46 Gy (RBE = 1.1)/23Fx was prescribed using the initial plan and 24 Gy (RBE = 1.1)/12Fx was prescribed using the adaptive plan.

Robust optimization was used to prescribe at least 70 Gy (RBE = 1.1) at D_99%_ of CTV by shifting the CT images by 3 mm and assuming an uncertainty in the range of 3.5%.

The dose constraints for OARs in the inverse planning with IMPT are shown in the right column of Supplementary Table S1 (Spinal cord PRV ≤50 Gy (RBE = 1.1), Brainstem PRV ≤60 Gy (RBE = 1.1), Optic nerves PRV ≤54 Gy (RBE = 1.1) and Optic chiasm PRV ≤54 Gy (RBE = 1.1)). Plans were optimized to ensure 100% of the prescribed dose to cover 99% of the volume of CTV46 and CTV70.

For the initial CT, a plan to irradiate 70 Gy (RBE = 1.1)/35Fx to CTV46 was developed (initial plan). The plan was created so that dose regulation for the target and OARs would meet the criteria. Next a plan to CTV70 with 70 Gy (RBE = 1.1)/35Fx was created on the second CT (adaptive plan), and the dose regulation for the target/OARs also meet the criteria. Finally the dose in the initial plan was multiplied by 46/70 and the doses in the adaptive plan was multiplied by 24/70, and the sum was regarded as the dose to the CTV and OARs.

### 2.5 Image registration, dose accumulation, and evaluation

**Fig. 1:**
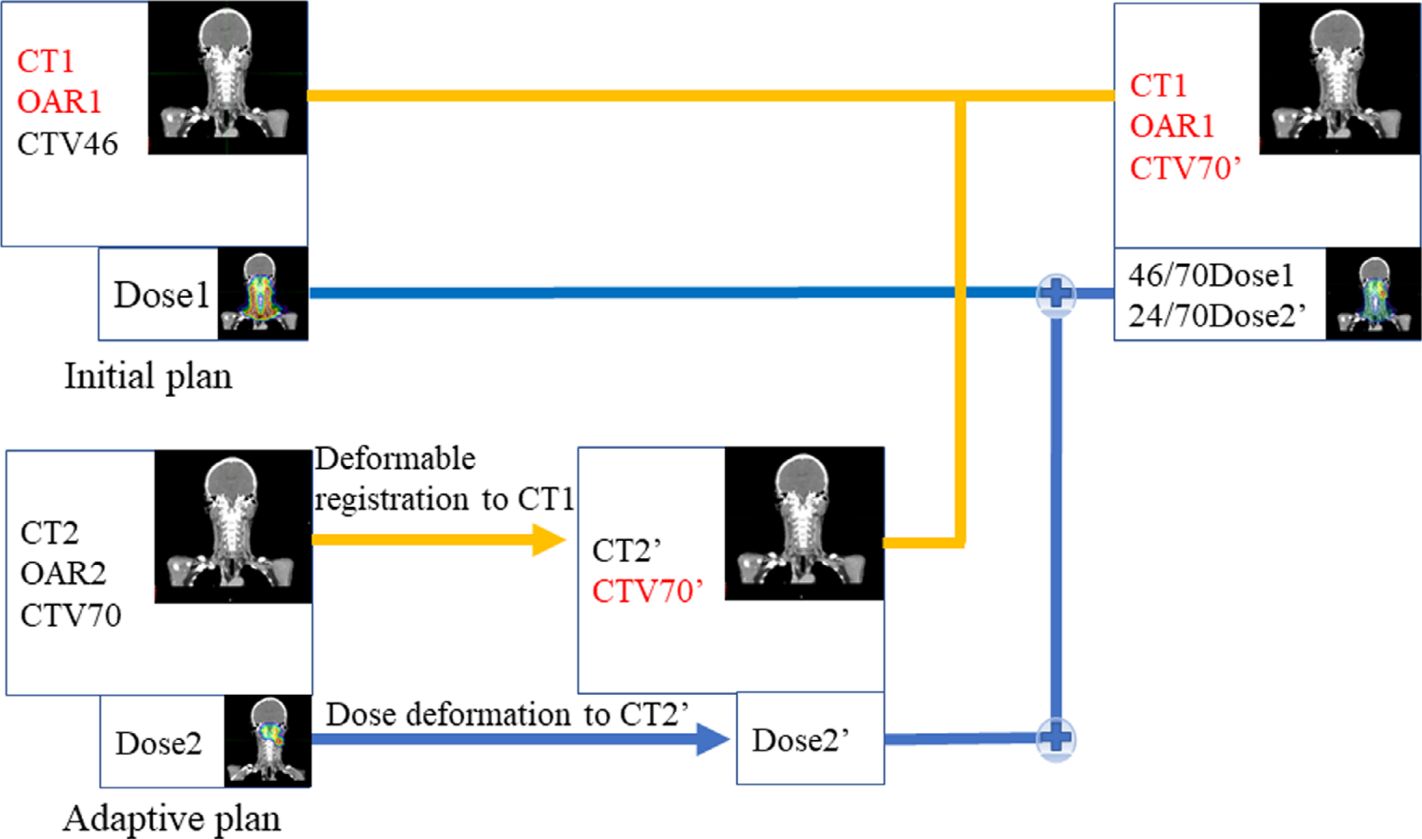
Schematic workflow for the dose accumulation procedure. The Blue line shows the flow of accumulation and deformation of the dose. The Yellow line shows the flow of deformation of the CT imaging, OAR, and CTV. CT1 = CT imaging used in initial plan. OAR1 = Organs at risk in initial plan. CTV46 = Clinical target volume in initial plan. Dose1 = Dose in initial plan. CT2 = CT imaging used in adaptive plan. OAR2 = OARs used in adaptive plan. CTV70 = Clinical target volume in adaptive plan. Dose2 = Dose in adaptive plan. CT2’ = CT imaging deformed to CT1. CTV70’= CTV which is deformed CTV70. Dose2’ = Dose which is the deformed Dose2 to fit CT2.

We decided to deform the adaptive plan CT to the original plan according to a previous study by Gora et al. which described adaptive radiotherapy for head and neck cancers.^25^ Our method of adaptive treatment planning and dose accumulation in A‐IMXT and A‐IMPT in this study was based on this method as is illustrated in Fig. 1. We used MIM version 6.92 (EuroMeditec, Tokyo) for the deformable image registration of the initial and adaptive plans. The CT images in the adaptive plan were deformed to be registered into the CT images in the initial plan. The CTV70 was deformed and registered into the CT used in the initial plan. The deformed CTV70 is denoted as CTV70’. Dose volume statistics were carried out by adding the dose in the initial plan to the dose in the adaptive plan on the CT images in the initial plan.

### 2.6 Statistical comparison

The conformation number (CN) published by van’t Riet et al. was used for the comparison between IMXT and IMPT.^26^ The CN is the product of a tumor coverage factor and a normal tissue over dosage factor. In this study, the CN is the product of CTV covered by the volume of 70 Gy divided by CTV, and CTV covered by the 70 Gy divided by the volume of the prescription isodose, that is, 70 Gy in the body.

The homogeneity index (HI) was obtained by dividing the dose administered by 5% of CTV70’ (D_5%_) by the dose which was administered to 95% of CTV70’ (D_95%_). Dose volume parameters were selected to be compatible with previous studies as far as possible. Dose volume statistics for the OARs were all analyzed and a comparison of A‐IMXT and A‐IMPT was made. The paired t‐test was used to compare the mean dose (D_mean_), the D_0.03cc_ and the maximum dose level given to each OAR. The dose for 30% (D_30%_) of the Eustachian tube and dose for 0.5cc of mastoid air cells volume (D_0.5cc_) were also measured since these have been reported to be important dose volume parameters associated with ear disorders following IMXT.^19^

## 3 RESULTS

### 3.1 Comparison of dose distribution

**Fig. 2:**
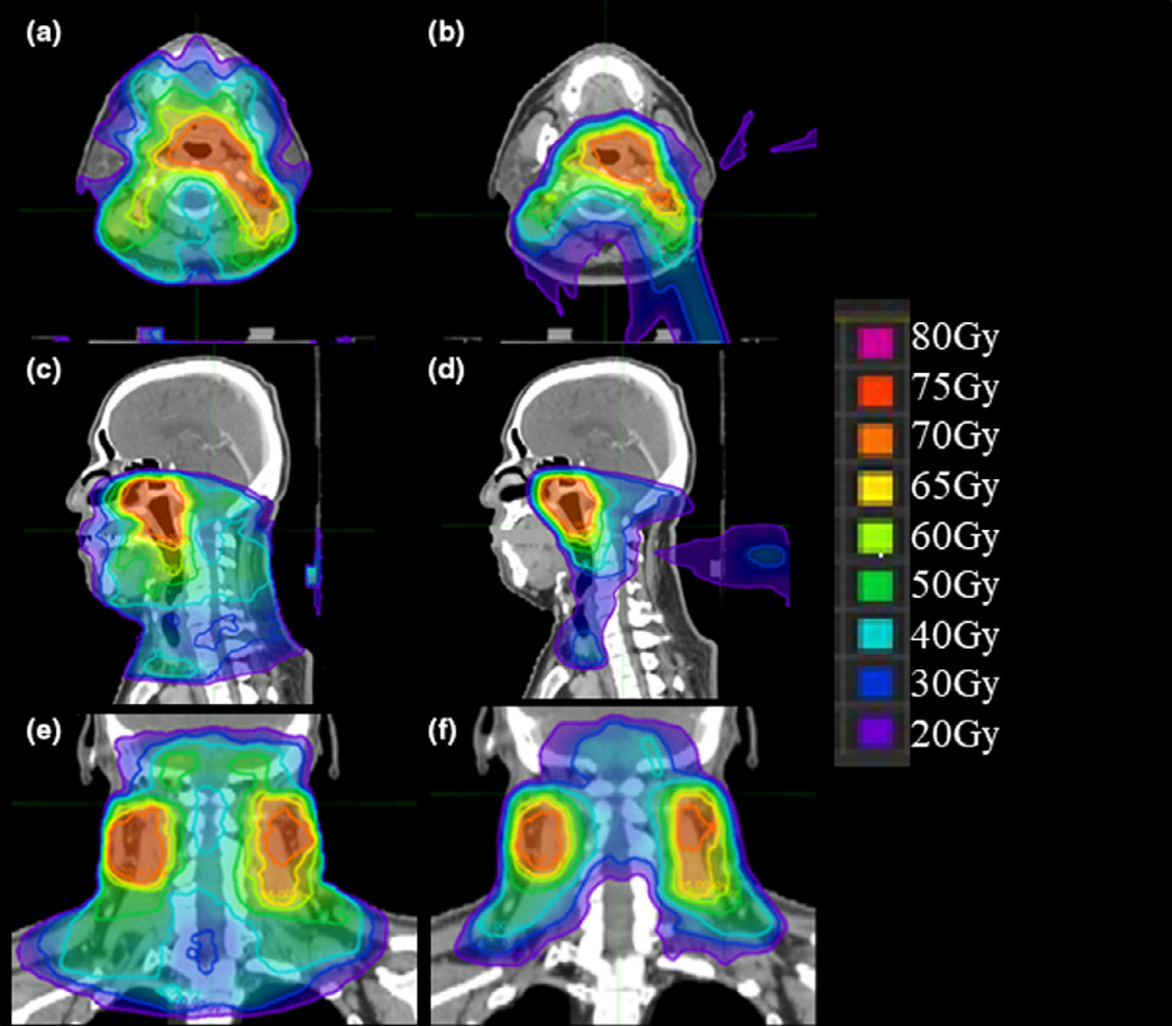
Representative treatment planning images for a patient with T1N2 stageⅢNPC. Isodose distributions of A‐IMXT (left) and A‐IMPT (right). In the axial plane for (a) A‐IMXT and (b) A‐IMPT plan. In the sagittal plane for (c) A‐IMXT and (d) A‐IMPT plan. In the coronal plan for (e) A‐IMXT and (f) A‐IMPT plan.

**Fig. 3:**
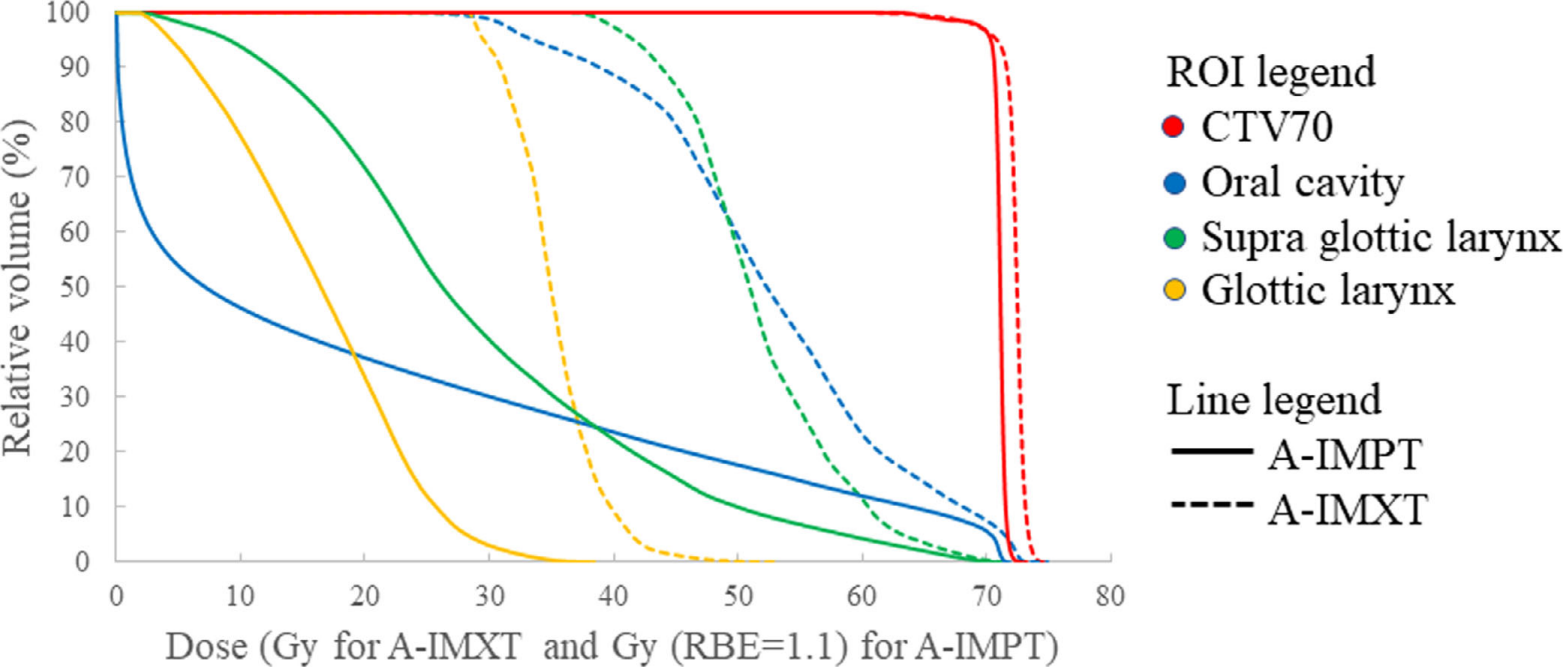
Dose volume histogram of A‐IMXT and A‐IMPT of the same patient as shown in Fig. 2.

Figures 2 and 3 show isodose distributions and the corresponding dose volume histogram (DVH), respectively, of A‐IMXT and A‐IMPT for the same NPC patient. Comparing the two dose distributions, it shows that the doses to the oral cavity and larynx in A‐IMPT are significantly lower than those in A‐IMXT. In A‐IMPT, the anterior part of the oral cavity and a part of the larynx received no irradiation although a low but definite dose was administered to the oral cavity and larynx in A‐IMXT.

### 3.2 CN and HI of CTV

There were no statistical differences in the mean V_70Gy_ (CTV70’) (*P* = 0.59), D_5%_ (CTV70’) (*P* = 0.149), and the D_95%_ (CTV70’) (*P* = 0.56) between A‐IMXT and A‐IMPT suggesting equivalence between the prescriptions for A‐IMXT and A‐IMPT (Table 2). However, the mean V_70Gy_ (BODY) (*P* < 0.001) was significantly lower in A‐IMPT than in A‐IMXT.

**Table 2:** Average dose statistics of the target of the A‐IMXT and A‐IMPT treatment plans.

Parameter	A‐IMXT (Mean ± SD)		A‐IMPT (Mean ± SD)		*P* value
V_70Gy_ (BODY)	392.3 ± 143.2	cc	207.0 ± 78.9	cc	<0.001
V_70Gy_ (CTV70’)	323.4 ± 132.1	cc	207.7 ± 80.7	cc	0.59
D_5%_ (CTV70’)	74.0 ± 0.7	Gy	71.4 ± 0.5	Gy (RBE = 1.1)	0.149
D_95%_ (CTV70’)	73.4 ± 1.1	Gy	71.6 ± 0.8	Gy (RBE = 1.1)	0.56
Conformation number	0.52 ± 0.06		0.64 ± 0.04		<0.001
Homogeneity index	1.04 ± 0.01		1.03 ± 0.01		0.00319

A‐IMXT, intensity‐modulated X‐ray therapy with adaptive approach; A‐IMPT, intensity‐modulated proton therapy with adaptive approach; SD, standard deviation; V_70Gy_, volume receiving 70 Gy; D_95%_, dose to the 95% of the volume; D_5%_, dose to the 5% of the volume.

There was a significant difference in the mean of the CN of A‐IMXT and A‐IMPT (0.52 ± 0.06 vs 0.64 ± 0.04, *P* < 0.001). The mean HI of CTV70’ in A‐IMPT (1.03 ± 0.01) was lower than the mean HI of A‐IMXT (1.04 ± 0.01), and was also statistically significant (*P* = 0.00319).

### 3.3 Dose volume statistics for the OARs

All parameters could be evaluated in all 10 patients. The means of the D_mean_ of the right parotid gland (*P* < 0.001), right TM joint (*P* < 0.001), left TM joint (*P* < 0.001), oral cavity (*P* < 0.001), supraglottic larynx (*P* < 0.001), glottic larynx (*P* < 0.001), middle PCM (*P* = 0.0371), interior PCM (*P* < 0.001), cricopharyngeal muscle (*P* = 0.00364), and thyroid gland (*P* = 0.00216) in A‐IMPT were lower than those with A‐IMXT with statistical significance (Table 3). The means of the D_0.03cc_ of the right optic nerve (*P* = 0.0136), spinal cord (*P* = 0.00492), brain stem (*P* = 0.00467), right TM joint (*P* < 0.001), and left TM joint (*P* < 0.001) in A‐IMPT are lower than those with A‐IMXT with statistical significance.

**Table 3:** Dose volume parameters for each OAR in A‐IMXT and A‐IMPT plans.

OARs	Parameter	A‐IMXT (Mean ± SD)	A‐IMPT (Mean ± SD)	*P*‐value
		Gy	Gy (RBE = 1.1)	
R parotid	D_mean_	30.6 ± 6.8	22.7 ± 7.2	<0.001
L parotid	D_mean_	34.9 ± 8.9	35.8 ± 9.8	0.63
R TM joint	D_mean_	38.4 ± 8.4	10.1 ± 11.5	<0.001
L TM joint	D_mean_	43.9 ± 13.5	32.2 ± 14.4	<0.001
Oral cavity	D_mean_	50.4 ± 3.8	21.7 ± 6.2	<0.001
Supraglottic larynx	D_mean_	45.5 ± 7.7	25.5 ± 8.6	<0.001
Glottic larynx	D_mean_	37.3 ± 6.8	15.3 ± 6.3	<0.001
Superior PCM	D_mean_	69.2 ± 3.7	66.7 ± 3.0	0.0624
Middle PCM	D_mean_	53.9 ± 10.0	45.7 ± 11.5	0.0371
Inferior PCM	D_mean_	42.0 ± 7.4	31.2 ± 7.1	<0.001
Cricophyarngeal muscle	D_mean_	37.5 ± 6.0	30.0 ± 6.3	0.00364
Thyroid gland	D_mean_	47.5 ± 6.8	40.1 ± 6.8	0.00216
R optic nerve	D_0.03cc_	24.8 ± 17.0	19.9 ± 15.2	0.0136
L optic nerve	D_0.03cc_	25.4 ± 19.3	22.8 ± 13.6	0.38
Chiasma	D_0.03cc_	18.1 ± 16.7	17.6 ± 14.4	0.70
Pituitary gland	D_0.03cc_	46.5 ± 23.4	43.0 ± 23.9	0.24
Brain stem	D_0.03cc_	55.4 ± 2.9	51.1 ± 4.7	0.00467
Spinal cord	D_0.03cc_	45.3 ± 1.6	42.7 ± 3.2	0.00492
R TM joint	D_0.03cc_	53.0 ± 9.7	25.7 ± 18.1	<0.001
L TM joint	D_0.03cc_	59.7 ± 13.1	48.3 ± 16.9	<0.001

A‐IMXT, intensity‐modulated X‐ray therapy with adaptive approach; A‐IMPT, intensity‐modulated proton therapy with adaptive approach; SD, standard deviation; R, right; L, left; TM joint, temporomandibular joint; PCM, pharyngeal constrictor muscle; D_mean_, mean dose; D_0.03cc_, dose to the highest 0.03cc area.

The means of the D_0.03cc_ and the D_mean_ of the sub portion of the left middle ear, right middle ear, and right inner ear in A‐IMPT were lower than in A‐IMXT with statistical significance individually as shown in Table 4. D_30%_ for Eustachian tube, D_0.5cc_ and D_0.03cc_ for mastoid air cells volume were all significantly lower in A‐IMPT comparing to A‐IMXT in both ears.

**Table 4:** Dose volume parameters for OARs related to hearing in A‐IMXT and A‐IMPT plans.

	OARs	Parameter	A‐IMRT (Mean ± SD)	A‐IMPT (Mean ± SD)	*P*‐value
L middle ear	L tympanic cavity	D_0.03cc_	57.3 ± 14.1	51.9 ± 17.8	0.0372
		D_mean_	49.2 ± 14.2	39.1 ± 18.6	0.00492
	L Eustachian tube	D_0.03cc_	62.1 ± 12.5	57 ± 15.9	0.00711
		D_mean_	56.6 ± 15.1	50.8 ± 17.0	0.00169
		D_30%_	61.2 ± 12.8	54.5 ± 17.1	0.00629
L mastoid air cells		D_0.03cc_	59.1 ± 13.4	48.8 ± 23.5	0.0118
		D_0.5cc_	51.6 ± 13.5	39.1 ± 21.8	0.00161
L inner ear	L cochlea	D_0.03cc_	52.1 ± 14.6	48.3 ± 18.2	0.0636
		D_mean_	51.1 ± 15.0	47.2 ± 18.4	0.0441
	L IAM	D_0.03cc_	50.4 ± 16.6	47.4 ± 19.2	0.21
		D_mean_	48.6 ± 15.7	45.2 ± 19.0	0.147
R middle ear	R tympanic cavity	D_0.03cc_	46.7 ± 11.9	31.7 ± 18.3	0.00224
		D_mean_	39.5 ± 11.9	20.6 ± 18.0	<0.001
	R Eustachian tube	D_0.03cc_	55.0 ± 11.7	43.7 ± 16.2	0.00472
		D_mean_	49.4 ± 10.2	35.7 ± 16.7	0.00257
		D_30%_	53.4 ± 10.9	41.7 ± 16.4	0.00769
R mastoid air cells		D_0.03cc_	55.3 ± 13.0	43.1 ± 18.4	0.00193
		D_0.5cc_	45.2 ± 12.3	28.6 ± 16.8	<0.001
R inner ear	R cochlea	D_0.03cc_	42.8 ± 10.9	32.3 ± 15.9	0.00343
		D_mean_	42.3 ± 10.8	31.7 ± 16.0	0.00325
	R IAM	D_0.03cc_	40.8 ± 11.9	32.9 ± 15.3	0.00807
		D_mean_	39.7 ± 12.3	31.5 ± 15.5	0.00455

A‐IMXT, intensity‐modulated X‐ray therapy with adaptive approach; A‐IMPT, intensity‐modulated proton therapy with adaptive approach; SD, standard deviation L, right; R, Right; IAM, internal auditory canal; D_0.03cc_, dose to the highest 0.03cc area; D_mean_, mean dose; D_30%_, dose to the 95% of the volume.

The means of the V_60Gy_ (*P* = 0.00228), V_50Gy_ (*P* = 0.00164), V_40Gy_ (*P* < 0.001), V_30Gy_ (*P* < 0.001), V_20Gy_ (*P* < 0.001), V_10Gy_ (*P* < 0.001), and V_5Gy_ (*P* < 0.001) to the skin in A‐IMPT are lower than those with A‐IMXT with statistical significance as shown in Table 5.

**Table 5:** Dose volume parameters for skin in A‐IMXT and A‐IMPT.

OAR	Parameter	A‐IMXT (Mean ± SD)	A‐IMPT (Mean ± SD)	*P*‐value
		Gy	Gy (RBE = 1.1)	
Skin	V_60Gy_	29.3 ± 29	22.4 ± 25.6	0.00228
	V_50Gy_	60.3 ± 48.5	37.3 ± 35.6	0.00164
	V_40Gy_	142.4 ± 72.1	81.3 ± 35.8	<0.001
	V_30Gy_	262.6 ± 72.2	129.3 ± 34.3	<0.001
	V_20Gy_	445.6 ± 56.0	208.2 ± 36.4	<0.001
	V_10Gy_	645.5 ± 46.7	349.9 ± 32.4	<0.001
	V_5Gy_	743.9 ± 58.5	434.3 ± 39.0	<0.001

A‐IMXT, intensity‐modulated X‐ray therapy with adaptive approach; A‐IMPT, intensity‐modulated proton therapy with adaptive approach; SD, standard deviation; V_5Gy_, volume receiving 5 Gy; V_10Gy_, volume receiving 10 Gy; V_20Gy_, volume receiving 20 Gy; V_30Gy_, volume receiving 30 Gy; V_40Gy_, volume receiving 40 Gy; V_50Gy_, volume receiving 50 Gy; V_60Gy_, volume receiving 60 Gy.

## 4 DISCUSSION

There have been several studies reporting comparisons between IMXT and IMPT for NPC: Taheri‐Kadkhoda et al.^11^ compared the IMPT and IMXT plans and showed that IMPT improves the tumor coverage and conformation and reduces the average mean dose to several organs at risk. In that study, the average mean dose to the oral cavity in IMPT (mean: min‐max, 38.1: 33.9–43.7) was significantly lower than with IMXT (44.0: 40.5–46.7). The average mean dose to the larynx in IMPT (14.3: 8.6–18.5) was also significantly lower than with IMXT (30.6: 24.3–35.3).

Widesott et al.^12^ compared IMPT and IMXT plans of helical tomotherapy for nasopharyngeal cancer, and found that IMPT plans showed lower average mean doses for the parotid glands and comparable average mean doses for the thyroid gland. They did not compare dose distributions of IMXT and IMPT to the oral cavity, but instead made a ROI of the “mucosa outside PTV” which includes the oral cavity and the pharynx, with a 0.5 cm margin added to the PTV. The average mean dose to the mucosa outside the PTV for IMRT was 26.2 ± 2.7 Gy and for IMPT it was 18.0 ± 1.6 Gy (RBE = 1.1). The mean dose for the pharyngeal constrictor muscle was not evaluated as an OAR. It was concluded that IMPT allows a better sparing of most OARs at medium‐to‐low doses.

Lewis et al.,^14^ in a study with nine NPC patients, compared IMXT and IMPT plans. They compared mean doses to 29 OARs reporting that the IMPT plans reduced 13 OAR mean doses significantly, over the doses of the IMXT plan. The mean dose to the oral cavity in IMPT (16.56 ± 7.67 Gy) was significantly lower than with IMXT (36.96 ± 7.90 Gy) in that study. The mean dose of the larynx in IMPT (29.56 ± 7.83 Gy (RBE = 1.1)) was also significantly lower than with IMXT (42.75 ± 9.94 Gy (RBE = 1.1)). The dose to the parotid glands were not significantly different. The doses to the thyroid gland and pharyngeal constrictor muscle were not evaluated.

None of these previous studies used adaptive approaches with either IMXT or IMPT. A‐IMXT for head and neck cancer has been reviewed recently by Castelli et al.^27^ Here it was found that further randomized clinical trials are required to show more solid evidence. Previous studies suggest benefits of A‐IMXT when comparing with IMXT in decreasing xerostomia, increasing quality of life and increasing local control. Patients with the largest early anatomical and dose variations were suggested to be the best candidates for A‐IMXT. The present study shows that the A‐IMPT plan would significantly increase the advantages of IMPT over IMXT for dose reductions to the OARs while maintaining tumor coverage.

We found that, compared with A‐IMXT, A‐IMPT reduces the D_mean_ to the right parotid gland, the oral cavity, thyroid gland, supraglottic larynx, glottic larynx, middle, inferior, cricopharyngeal pharyngeal constrictor muscle, right and left TM joints, the D_0.03cc_ to the brain stem, spinal cord, right optic nerve and right and left TM joints, while it maintains optimal tumor coverage for NPC significantly better. Notably, the differences in the mean dose to the oral cavity and larynx were much larger than the differences between IMXT and IMPT without the adaptive approach as reported in previous studies by Taheri‐Kadkhoda et al.,^11^ Widesott et al.^12^ and Lewis et al.^14^ In the present study, the mean doses to the oral cavity, supraglottic larynx, and glottic larynx were all reduced more than 20 Gy (RBE = 1.1) as shown in Table 3. In contrast, only one of the mean dose reductions to the OAR was larger than 20 Gy (RBE = 1.1) in the previous studies. Further, while there was no difference between IMXT and IMPT to the D_0.03cc_, or D_max_ to the brainstem, TM joints, and pituitary gland in the Taheri‐Kadkhoda et al. study, there was a significant difference between A‐IMXT and A‐IMPT in this study.

Yao et al. have recently reported that D_30%_ to the Eustachian tube >52.75 Gy and D_0.5cc_ to the mastoid air cells >41.04 Gy were associated with Grade 2 ear disorders in IMRT for NPC.^19^ We have found that A‐IMXT can reduce the D_30%_ to the Eustachian tube from 61.2 ± 12.8 Gy to 54.5 ± 17.1 Gy in the left side and from 53.4 ± 10.9 Gy to 41.7 ± 16.4 Gy in the right side. Also, the D_0.5cc_ to the mastoid air cells from 51.6 ± 13.5 Gy to 39.1 ± 21.8 Gy in the left side and from 45.2 ± 12.3 Gy to 28.6 ± 16.8 Gy in the right side. The mean values of the D_30%_ to the Eustachian tube in the right side and D_0.5cc_ to the mastoid air cells in both sides in the A‐IMPT are in the range where the incidence of Grade 2 ear disorders would be reduced significantly. Hearing impairment after radiotherapy for NPC is one of the most important adverse effects to be reduced by the new technology. The present study suggests that the incidence of Grade 2 ear disorders after radiotherapy for NPC may well be reduced by using A‐IMPT instead of A‐IMXT from the view point of dose volume statistics.

Taheri‐Kadkloda et al. also reported that the mean D_max_ to the skin was almost equal for IMXT and IMPT (66.8 Gy and 65.7 Gy (RBE = 1.1)) but the mean D_mean_ was significantly lower in the IMPT plan (9.6 Gy and 5.7 Gy (RBE = 1.1)). The appropriate dose volume parameter for skin reaction was recently reported to be V_5Gy_ to V_60Gy_ by Kawamura et al.^24^ We have found that A‐IMPT can reduce any of these dose parameters in this study.

These results imply that the adaptive approach enhances the potential benefit of IMPT over that of IMXT to reduce adverse effects significantly. Additionally, we found that the CN was significantly improved with A‐IMPT as compared with A‐IMXT for NPC in this study. Considering that the adaptive approach is generally effective to improve the CN of CTV for tumors which commonly change in shape of the CTV during radiotherapy,^28^ A‐IMPT has potential benefits to improve the therapeutic ratio for nasopharyngeal carcinomas.

The present study has several potential shortcomings: First, we have not compared the differences in normal tissue complication probabilities and tumor control probability for A‐IMXT and A‐IMPT. However, the differences in the mean dose to OARs at low dose levels and hot spots in the CTV may not lead to meaningful differences in clinical outcomes. It will be important to investigate the actual differences in adverse effects for each OARs in clinical outcomes in prospective clinical research.

A second shortcoming is the absence of established ways to estimate the appropriateness of A‐IMXT plans in our study in comparison to A‐IMPT plans. However, since A‐IMXT was used for the ten patients in our institution, there was no intention to make the A‐IMXT dose distribution suboptimal in this study. Thus, the superiority in the A‐IMPT plan in this study can be interpreted with more confidence than in studies using A‐IMXT plans which are made for virtual comparisons but not implemented clinically.

Third, we conducted the A‐IMXT planning using the step and shoot approach. Recently, however, the VMAT approach is more often used clinically. If the VMAT approach had been used instead of step and shoot, the difference for OARs between A‐IMXT and A‐IMPT may have been smaller. Ning et al.^29^ compared 20 patient dose distributions with single arc VMAT, dual arc VMAT and step and shoot IMRT. There the mean doses to the oral cavity were 40.35 ± 4.77 Gy, 36.99 ± 6.18 Gy, and 43.21 ± 5.60 Gy, respectively. We cannot simply compare these results with the present study, but assume that even if we make the IMXT plan with the VMAT technique, the dose to the oral cavity in the A‐IMPT plan would have been preferential to the adaptive VMAT plan. This is because low but definite doses are irradiated to the entire oral cavity in the VMAT plan in the Ning et al.^29^ study while the anterior part of the oral cavity was spared from any irradiation in the A‐IMPT plan in all the patients in this study.

As a future study, we are considering to add an indicator for re‐planning such as 5% weight loss in addition to the fixed adaptation course. Jiri et al. have recently published initial clinical results of IMPT for NPC.^30^ They used an individualized adaptive course that triggered the new plan when the target volume change reached some threshold, rather than a fixed fraction number. A larger than 5% change in dose inside the critical organ or target volume was an indicator for re‐planning. The problem of this individualized approach would be the reproducibility of the treatment protocol in the particular institution. Alternatively, we may use a 2‐adaptation course rather than a 1‐adaptation course with IMPT as an alternative approach to keep the robustness of the treatment protocol. In either case, we will be able to use the present study as the basic dose volume data for A‐IMPT data to be compared.

## 5 Conclusions

Compared with A‐IMXT, A‐IMPT presents significant benefits in the dose distribution and potential benefits in reducing clinical adverse effects on OARs and with a better conformation number and superior dose homogeneity in the CTV of NPC. The adaptive approach is suggested to enhance the potential benefits of IMPT compared to IMXT by reducing adverse effects and by improving local control for patients with NPC. Further clinical studies are required to ensure the validity of the findings in this study.

## Conflict of interests

The authors have no conflict of interests to declare.
