# Supplementary material for: Potential benefits of adaptive intensity‐modulated proton therapy in nasopharyngeal carcinomas
Source: J Appl Clin Med Phys. 2020 Dec 18;22(1):174–83. doi: 10.1002/acm2.13128 (PMC7856494; doi:10.1002/acm2.13128)
Supplement: Supplementary file 1 [file ACM2-22-174-s001.docx]

**Supplementary table 1**

The objective parameters used in IMXT and IMPT optimization

| Target and risk organs | Dose constrains(IMXT) | Dose constrains(IMPT) |
| --- | --- | --- |
| PTV(IMRT), CTV(IMPT) | D_95%_=70Gy | D_99%_=70Gy(RBE=1.1) |
|  | D_98%_≥65.1Gy | D_98%_≥65.1Gy(RBE=1.1) |
|  | D_95%_=70Gy |  |
|  | D_50%_≤73.5Gy | D_50%_≤73.5Gy(RBE=1.1) |
|  | D_15%_≤77Gy | D_15%_≤77Gy(RBE=1.1) |
|  | D_max_≤84% | D_max_≤84Gy(RBE=1.1) |
| Spinal cord (PRV) | D_max_≤50Gy | D_max_ ≤50Gy (RBE=1.1) |
| Brain stem (PRV) | D_max_≤60Gy | D_max_≤60Gy (RBE=1.1) |
| Right optic nerve (PRV) | D_max_≤54Gy | D_max_≤54Gy (RBE=1.1 |
| Left optic nerve (PRV) | D_max_≤54Gy | D_max_≤54Gy (RBE=1.1) |
| Chiasma (PRV) | D_max_≤54Gy | D_max_≤54Gy (RBE=1.1) |
| Right parotid gland | either  D_mean_≤26Gy | either D_mean_≤26Gy(RBE=1.1) |
| Left parotid gland |  |  |
| Oral cavity | As low as possible | As low as possible |
| Thyroids gland | As low as possible | As low as possible |
| Larynx | As low as possible | As low as possible |

PTV, Planning target volume; PRV, Planning organ at risk volume; D_max_, maximum dose; D_mean_, mean dose; D_99%_, dose to the 99% of the volume; D_98%_, dose to the 98% of the volume; D_95%_, dose to the 95% of the volume; D_50%_, dose to the 50% of the volume D_15%_, dose to the 15% of the volume.
